# Supplementary material for: Ganoderma lucidum (Curtis) P. Karst. Immunomodulatory Protein Has the Potential to Improve the Prognosis of Breast Cancer Through the Regulation of Key Prognosis-Related Genes
Source: Pharmaceuticals (Basel). 2024 Dec 16;17(12):1695. doi: 10.3390/ph17121695 (PMC11677753; doi:10.3390/ph17121695)
Supplement: Supplementary file 1 [file pharmaceuticals-17-01695-s001.zip › pharmaceuticals-3279023-supplementary.pdf]

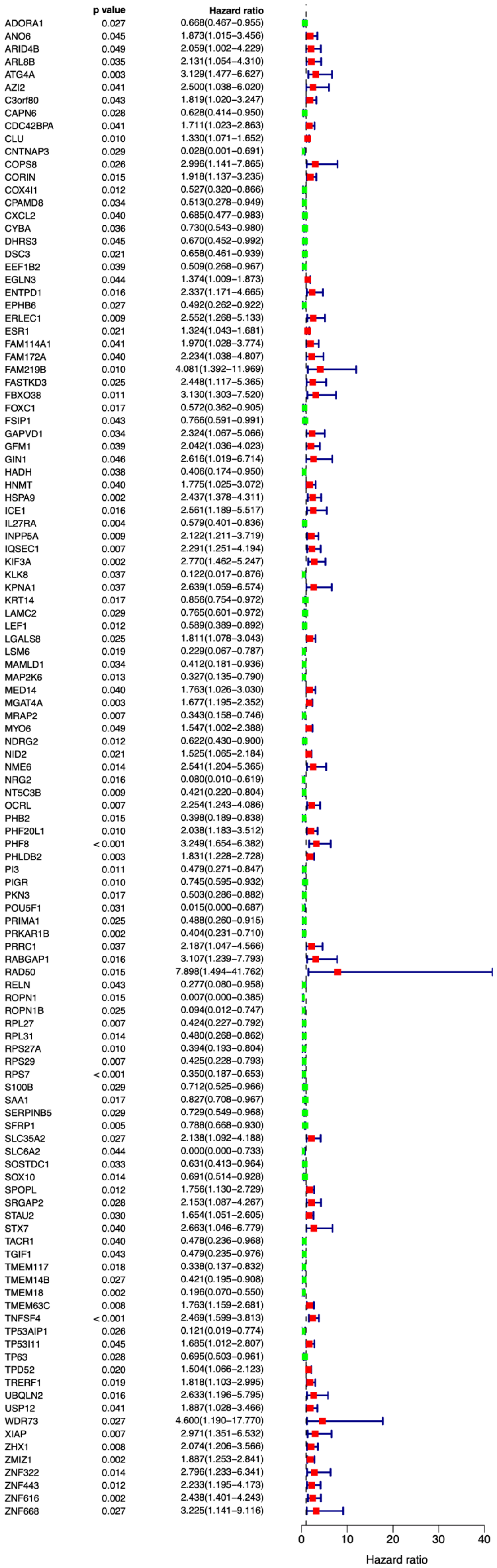

Figure S1. Forest plot showed the prognostic risk value of the 117 screened mRNAs by univariate Cox regression analysis.

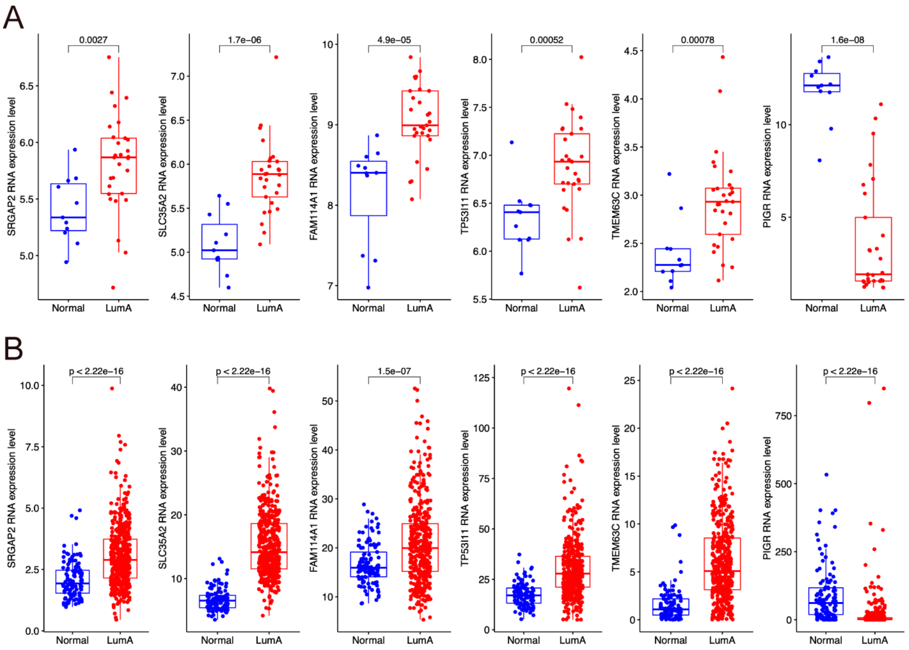

Figure S2. Boxplot diagram of the expression of risk mRNAs in normal and LumA BC tissues from GSE42568 (A) and TCGA (B).

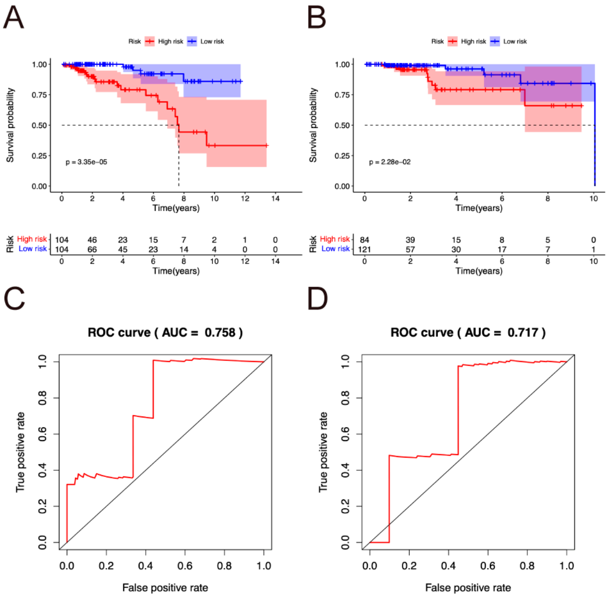

Figure S3. Survival analysis of the model in the training and test sets. (A) The Kaplan–Meier survival analysis of OS between the high-risk group and low-risk group in the training set. (B) The Kaplan–Meier survival analysis of OS between the high-risk group and low-risk group in the test set. (C) AUC in ROC analysis for risk signature at 1-year survival time in the training set. (D) AUC in ROC analysis for risk signature at 1-year survival time in the test set.

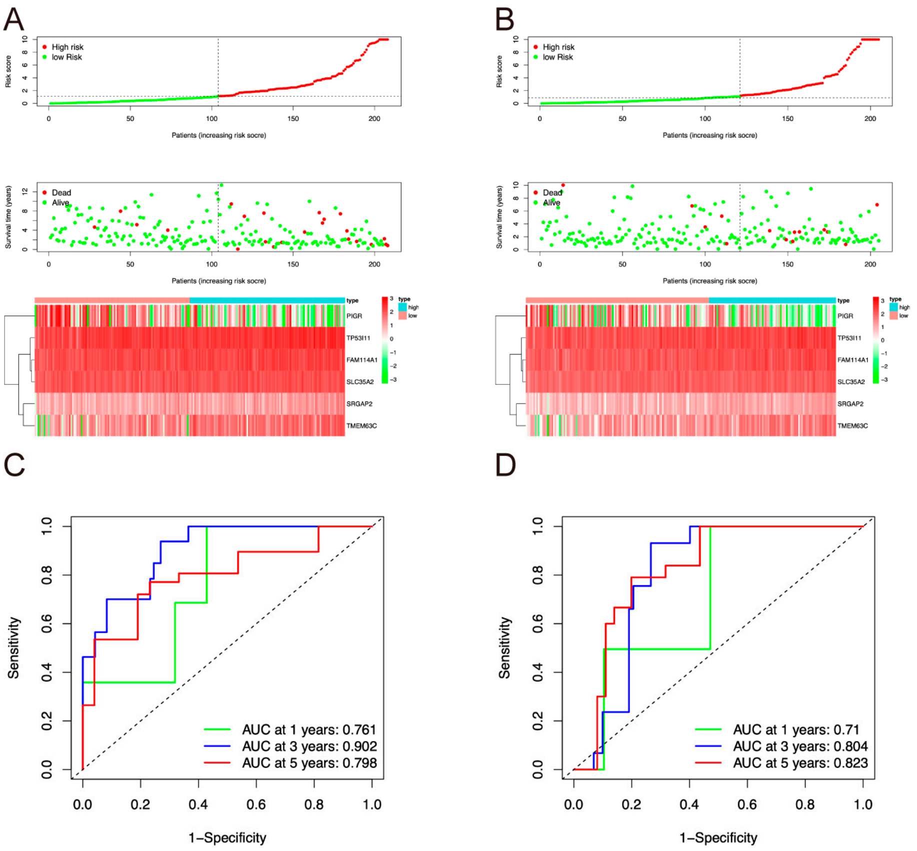

Figure S4. Construction of the prognostic risk model. Distribution of the risk score and the expression of risk genes in TCGA training set (A) and TCGA test set (B). Time-dependent ROC curve analysis of the risk score model for predicting 1-, 3-, and 5-year OS in TCGA training set (C) and TCGA test set (D).

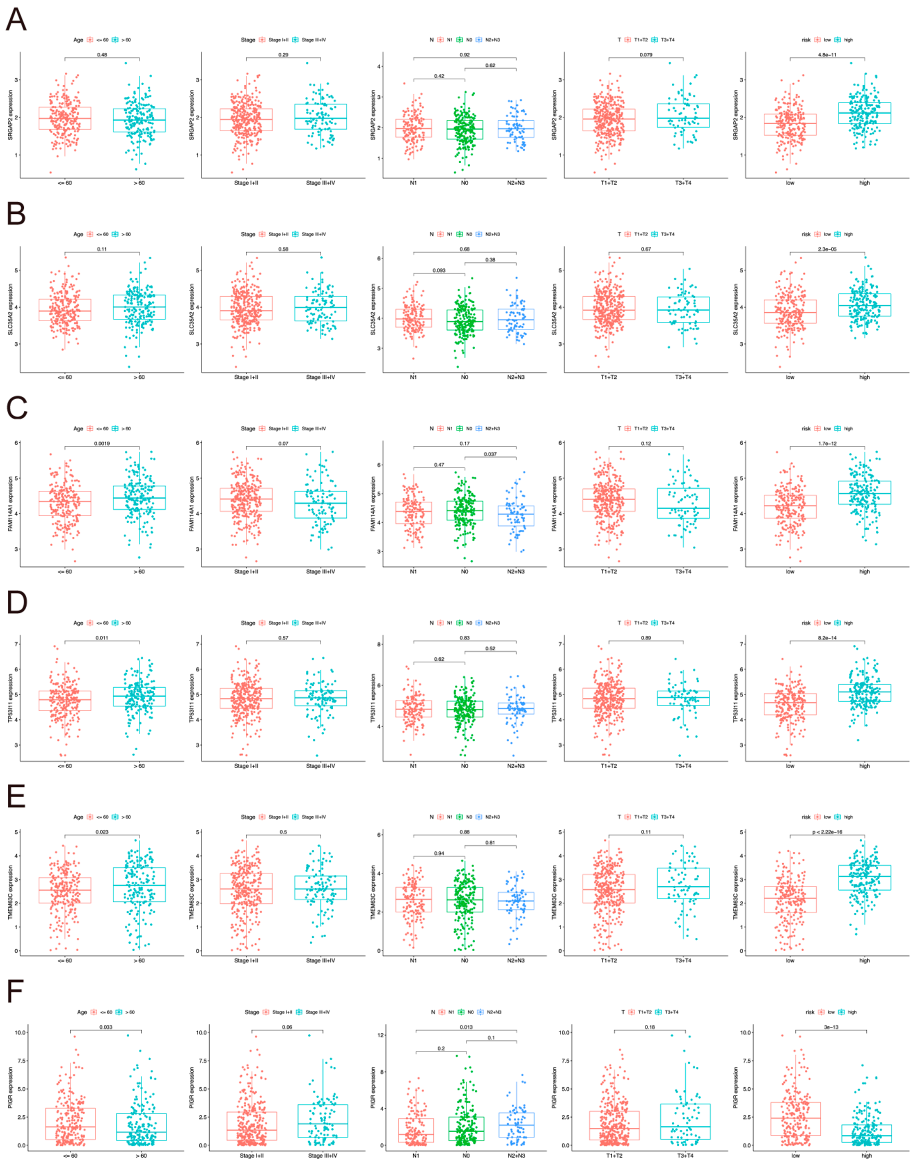

Figure S5. Boxplot diagram of the expression of risk genes SRGAP2 (A), SLC35A2 (B), FAM114A1 (C), TP53I11 (D), TMEM63C (E), and PIGR (F) in patients classified into the high-risk and low-risk groups based on the median risk score according to age, stage, N stage, and T stage.

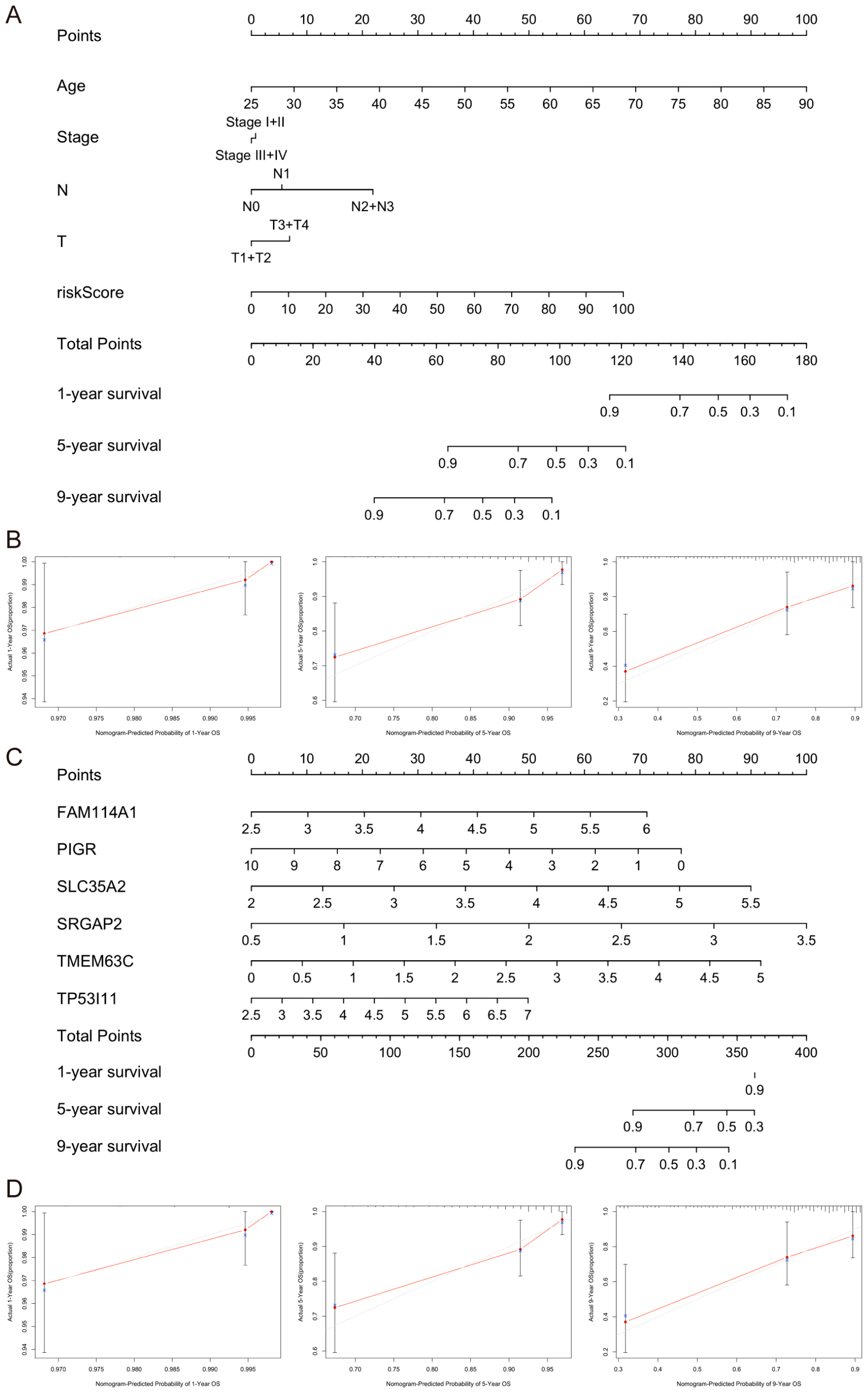

Figure S6. Establishment and evaluation of nomograms. (A) Construction of a nomogram incorporating prognostic model and clinical features including age, stage, N stage, and T stage. (B) Calibration plots for the nomogram (A) on 1-, 5-, and 9-year survival probability in patients with LumA BC. (C) Construction of a nomogram using the expression of model genes including SRGAP2, SLC35A2, FAM114A1, TP53I11, TMEM63C, and PIGR. (D) Calibration plots for the nomogram (C) on 1-, 5-, and 9-year survival probability in patients with LumA BC.

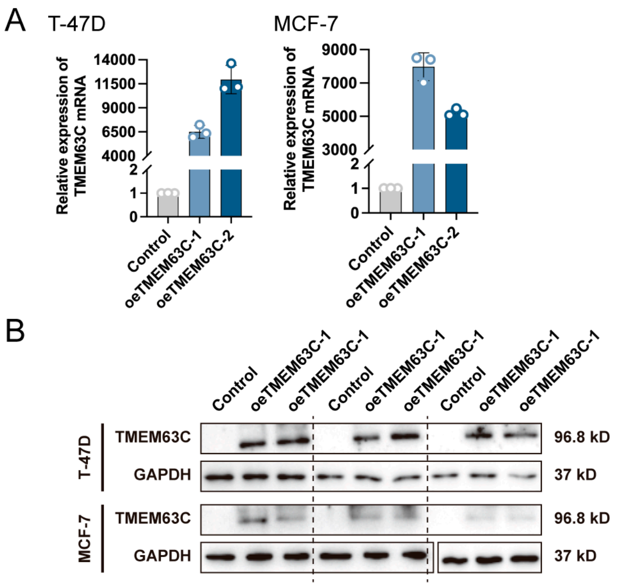

Figure S7. Validation of TMEM63C in breast cancer T-47D and MCF-7 cells detected by RT-qPCR (A) and Western blot (B).

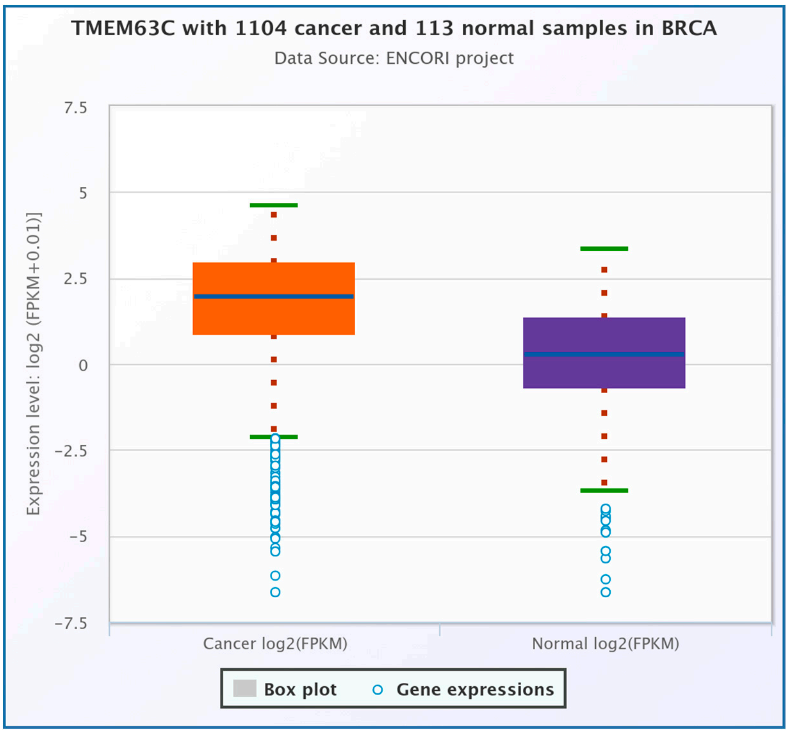

Figure S8. TMEM63C RNA level in TCGA from ENCORI.
